# Between burnout and resilience: a qualitative exploration of emotional labor among emergency department nurses in Saudi Arabia

**DOI:** 10.1186/s12912-026-04877-5

**Published:** 2026-06-15

**Authors:** Abdulaziz M. Alodhailah, Bader M. Almutairy, Faihan F. Alshaibany, Waleed M. Alshehri, Thurayya Eid

**Affiliations:** 1 Department of Medical-Surgical Nursing, College of Nursing, King Saud University, Riyadh, 11451 Saudi Arabia; 2 Department of Community and Psychiatric Mental Health Nursing, College of Nursing, King Saud University, Riyadh, 11451 Saudi Arabia; 3 Department of Nursing Administration and Education, College of Nursing, King Saud University, Riyadh, 11451 Saudi Arabia

**Keywords:** Burnout, Professional, Resilience, Psychological, Emotional labor, Emergency nursing, Saudi Arabia

## Abstract

**Background:**

Emergency department (ED) nurses manage intense emotional labor that contributes to burnout while simultaneously shaping resilience. Although burnout is a recognized hazard, the dynamic interplay between burnout and resilience within the Saudi Arabian healthcare context remains poorly understood. This study explored how ED nurses experience emotional labor and the relationship between burnout and resilience.

**Methods:**

A qualitative descriptive study was conducted with 17 ED nurses purposively sampled from three healthcare facilities in Riyadh, Saudi Arabia. Semi-structured interviews, lasting 38–67 min, were conducted between December 2025 and February 2026. Data were analyzed using reflexive thematic analysis, informed by emotional labor and Conservation of Resources theories. Trustworthiness was ensured through member checking, peer debriefing, and an audit trail. Reporting followed COREQ guidelines.

**Results:**

Five themes were generated: (1) demands of emotional labor, including high-acuity care and emotional suppression; (2) manifestations of burnout, such as exhaustion and depersonalization; (3) resilience mechanisms, comprising peer support and professional meaning-making; (4) organizational influences, involving leadership quality and resource adequacy; and (5) participant-generated recommendations for systemic reform. Burnout and resilience were experienced as concurrent, dynamic processes modulated by the organizational environment.

**Conclusions:**

Burnout and resilience coexist as concurrent dynamic processes in Saudi ED nurses, with organizational factors, staffing adequacy, leadership quality, and mental health support, acting as decisive determinants of occupational wellbeing. Strengthening organizational accountability, rather than individual adaptation alone, is essential for sustainable emergency nursing practice.

## Background

Emergency departments are among the most psychologically demanding clinical environments in modern healthcare, characterized by unpredictable patient acuity, complex triage decisions under time pressure, frequent exposure to traumatic presentations, and sustained interaction with distressed patients and their families [1, 2]. These conditions generate substantial emotional labor, defined by Hochschild [3] as the management of feelings and their outward expression to fulfil organizational role requirements. When emotion regulation demands are sustained without adequate organizational recognition or support, nurses become vulnerable to professional burnout [4, 5].

Professional burnout, conceptualized by Maslach and Leiter [6] as a syndrome of emotional exhaustion, depersonalization, and reduced personal accomplishment, represents a significant occupational hazard for emergency nurses worldwide. Meta-analytic evidence indicates burnout prevalence rates of 31–54% among ED nurses internationally [7, 8], with consequences extending beyond individual wellbeing to encompass diminished patient safety, increased medication errors, elevated workforce attrition, and degraded quality of care [9–11]. Within the Middle Eastern region, emerging data suggest comparable or higher prevalence [12, 13], yet context-specific qualitative evidence remains notably sparse.

Complementarily, resilience, understood as the dynamic capacity to adapt and sustain psychological functioning despite significant adversity, has attracted growing research attention as a potentially modifiable counterbalance to burnout [4]. Contemporary resilience theory conceptualizes resilience not as a fixed individual trait but as a process shaped by ongoing interactions among personal capacities, interpersonal relationships, and organizational contexts [14, 15]. Within nursing, mechanisms including peer support, professional identity, meaning-making, and coping flexibility have been identified as sustaining engagement and career longevity [16, 17]. However, the specific manifestations and facilitators of resilience within ED nursing in Saudi Arabia remain underexplored.

Two complementary theoretical frameworks inform this investigation. Hobfoll’s Conservation of Resources (COR) theory [18] posits that stress and burnout arise from actual or threatened resource loss, and that resilience is sustained through resource gain and protection across personal, social, and organizational domains. Hochschild’s emotional labor theory [3] illuminates how specific emotion regulation demands, including surface acting (displaying emotions not genuinely felt), deep acting (modifying internal feelings to align with display requirements), and emotional suppression, progressively deplete psychological resources when organizational structures fail to provide adequate support. Together, these frameworks suggest that burnout and resilience are not dichotomous endpoints but overlapping, dynamic processes whose trajectories are modulated by the balance between occupational demands and available resources at multiple ecological levels.

The Saudi Arabian healthcare context shapes emotional labor and burnout through three culturally specific features. First, prevailing collectivist values strengthen peer solidarity but also intensify the social expectations placed on nurses to absorb and contain group distress. Second, the normative expectation of family involvement in healthcare creates a dual emotional labor burden: nurses simultaneously manage the patient’s clinical condition and the family system’s emotional response. Third, the centrality of Islamic faith and spiritual practice provides coping resources that function differently from the secular strategies on which most Western burnout interventions are premised [12, 19–21]. 

While quantitative studies have documented burnout prevalence in the Saudi region [12, 13], qualitative research offering nuanced understanding of how ED nurses experience, interpret, and navigate the burnout–resilience continuum within this specific cultural and organizational context is limited. This study addresses that gap by exploring the lived experience of emotional labor, burnout, and resilience among ED nurses in Saudi Arabia and by identifying modifiable factors that may inform targeted interventions. This study makes three distinct contributions. Theoretically, it challenges the conventional dichotomy between burnout and resilience by demonstrating their concurrent coexistence as dynamic processes. Contextually, it extends the qualitative evidence base to a non-Western, collectivist, and religiously embedded healthcare setting. Practically, it generates a multi-level intervention framework grounded in the direct experiences of Saudi ED nurses.

### Study aim

This study aimed to: (1) explore how ED nurses in Saudi Arabia experience and manage emotional labor in their daily practice; (2) describe the manifestations of burnout and resilience and how they are experienced concurrently within this population; and (3) identify organizational, interpersonal, and personal factors that influence burnout–resilience dynamics, with implications for intervention.

## Methods

### Study design and philosophical underpinnings

A qualitative descriptive design [22] employing semi-structured interviews was adopted. The study was situated within a constructivist epistemological framework, which recognizes that knowledge is co-constructed through researcher–participant interaction and that participants’ accounts are interpreted within the contexts that give them meaning [23]. Data were analyzed using Braun and Clarke’s [24, 25] reflexive thematic analysis, an approach that emphasizes the researcher’s active, reflexive role in pattern identification while maintaining systematic rigor. This approach was selected for its theoretical flexibility, its capacity to accommodate pre-existing theoretical frameworks alongside inductive engagement with data, and its suitability for producing rich, patterned accounts across a relatively homogeneous participant group.

The study was informed by two complementary theoretical lenses: Hochschild’s emotional labor theory [3] and Hobfoll’s Conservation of Resources theory [18]. These frameworks guided the development of the interview schedule, provided sensitizing concepts during analysis, and offered an interpretive lens for situating findings within established theoretical discourse, while maintaining openness to participants’ own constructions of meaning.

This study is reported in accordance with the Consolidated Criteria for Reporting Qualitative Research (COREQ) 32-item checklist [26].

### Setting

The study was conducted across three healthcare facilities in Riyadh, Saudi Arabia: two government-operated general hospitals and one specialized tertiary medical center. These facilities were purposively selected to provide organizational diversity in ED size, patient acuity profiles, and resourcing levels, thereby enhancing the range of occupational contexts represented in the data.

### Participants and sampling

Eligible participants were registered nurses who: (a) held current employment in an ED at one of the three participating facilities; (b) had accumulated a minimum of one year of ED nursing experience; (c) were fluent in Arabic or English; and (d) were able to provide informed consent. Nurses occupying exclusively administrative roles without direct patient care responsibilities were excluded.

Purposive sampling was employed to recruit a diverse group reflecting variation in age, gender, years of experience, educational background, and facility type [27]. Recruitment was facilitated through posted study announcements displayed in ED staff areas and through nurse manager referrals; to minimize perceived coercion, interested nurses contacted the research team directly and all subsequent communication occurred without manager involvement. Sample size was determined through ongoing assessment of information power [28]. Information power was evaluated across five dimensions: (1) sample specificity, participants shared a narrow, homogeneous clinical role as ED nurses in Riyadh facilities; (2) theoretical focus, the dual theoretical framework provided clear sensitizing concepts bounding the analytical scope; (3) interview quality, all 17 interviews were substantive (mean duration 47 min) and generated sufficient narrative depth; (4) cross-case analytical strategy—systematic comparison enabled identification of both shared patterns and meaningful variation; and (5) data sufficiency—recruitment ceased when two consecutive interviews (participants 16 and 17) introduced no substantively new analytical categories. Of 23 nurses who expressed initial interest, 19 met eligibility criteria and 17 consented and completed interviews; two eligible nurses declined due to scheduling constraints.

### Data collection

Semi-structured, individual interviews were conducted between December 2025 and February 2026 by the first author, a nursing academic with doctoral-level training in qualitative research methods and seven years of clinical experience in emergency nursing. An interview guide was developed from the existing literature and structured around five domains: (1) general ED experience, (2) emotional labor demands and burnout challenges, (3) coping strategies and resilience, (4) influencing factors including team, organizational, and systemic variables, and (5) recommendations for improvement. The guide comprised 20 open-ended questions with probing prompts designed to elicit depth and specificity (Additional File 2). The guide was pilot-tested with two ED nurses (not included in the final sample) and refined for clarity and flow.

Of the 17 interviews, 12 were conducted in Arabic and five in English, according to participant preference. All interviews took place in private rooms within the healthcare facilities, ensuring auditory privacy. Interviews lasted between 38 and 67 min (mean: 47 min; SD: 8.2 min). All interviews were audio-recorded with participant permission and transcribed verbatim. Arabic-language transcripts were translated into English by the first author (AMA), a bilingual researcher, and independently verified for semantic equivalence by the second author (BAM), also a native Arabic speaker with advanced English proficiency. Discrepancies were resolved through consensus discussion. Because both translators were native Arabic speakers with advanced English proficiency, consensus verification between independent translations was deemed a sufficient safeguard against translation error, and formal back-translation was not employed. All quotations derive from verified English translations. No systematic thematic differences were observed between participants interviewed in Arabic and those interviewed in English.

Field notes documenting non-verbal communication, emotional affect, and contextual observations were recorded within one hour of each interview and were integrated into the analytical process as supplementary data.

### Data analysis

Data were analyzed using Braun and Clarke’s [24, 25] six-phase reflexive thematic analysis framework:

#### Phase 1: Familiarization

All transcripts were read in their entirety at least three times by the first author, with initial annotations and reflexive notes recorded during each reading. Audio recordings were re-listened to during the first reading to preserve tonal and paralinguistic nuances.

#### Phase 2: Coding

Systematic line-by-line coding was conducted on all 17 transcripts. To establish analytical dialogue and coding consistency, two researchers (AMA and BAM) independently coded three transcripts and met to compare, discuss, and refine the coding approach. Subsequent coding was conducted by AMA using NVivo 14 software (Lumivero, Denver, CO, USA), with regular team discussions at each analytical phase. This process generated 234 initial codes.

#### Phase 3: Theme construction

Initial codes were clustered into candidate themes through iterative comparison and pattern identification. Codes were consolidated from 234 initial codes to 87 focused codes, then organized into 35 provisional subthemes grouped under candidate theme headings. 

#### Phase 4: Theme review

Candidate themes were reviewed against both the coded data extracts and the full dataset to ensure internal coherence and external distinctiveness. Themes were refined, merged, or reorganized; provisional subthemes were consolidated where overlap was identified.

#### Phase 5: Theme definition

Final themes were named and defined, with clear scope boundaries, constituent subthemes, and exemplar quotations specified for each.

#### Phase 6: Writing

The analytical narrative was produced, integrating descriptive findings with interpretive analysis and theoretical integration.

Analytical memos documenting coding rationales, interpretive decisions, and emergent patterns were maintained throughout all phases. A comprehensive code-generation tracking log demonstrated that new code production declined substantially after interview 12, with no new themes generated after interview 15 (Additional File 4).

### Researcher reflexivity

All five team members hold doctoral qualifications in nursing and possess clinical experience, with three having worked in ED settings. This insider positionality conferred advantages, as familiarity with ED terminology, workflows, culture, and emotional demands facilitated participant rapport and enabled contextually informed probing during interviews. However, shared professional socialization also risked over-identification with participant narratives and normalization of occupational distress that warranted critical analytical interrogation.

To manage these risks, several reflexive strategies were employed. The interviewer (AMA) maintained a reflexive journal throughout data collection and analysis, documenting preconceptions, emotional responses to interview content, evolving interpretations, and instances where personal experience may have influenced analytical decisions. Regular peer debriefing sessions (held fortnightly during data collection and weekly during analysis) provided structured opportunities for team members to challenge one another’s interpretations, identify areas of assumed consensus, and explore alternative readings of the data. The team deliberately sought disconfirming evidence and negative cases during analytical phases 3 and 4. A detailed reflexivity statement is provided in Additional File 3.

### Trustworthiness

Four strategies were employed following Lincoln and Guba’s [19] framework:

*Credibility* was enhanced through member checking, in which six participants (35% of the sample) reviewed preliminary theme descriptions and representative quotations. Feedback was solicited on theme naming, organisational structure of the thematic framework, and the interpretive approach. Participants confirmed the findings resonated with their experiences; minor refinements to theme language were incorporated. One participant queried the framing of ‘emotional suppression’ as distinct from professional composure, prompting clarification of scope boundaries in the theme definition; this feedback was adopted. No participant feedback was withheld without documented rationale. Peer debriefing also occurred through regular research team discussions throughout the analytical process.

*Transferability* was supported through provision of thick description of the research setting, participant characteristics, recruitment procedures, and contextual factors, enabling readers to assess the applicability of findings to other settings and populations.

*Dependability* was established through maintenance of a comprehensive audit trail documenting all analytical decisions, code development, theme evolution, and team discussion outcomes. The audit trail is summarized in Additional File 4 and is available in full from the corresponding author upon request.

*Confirmability* was supported by grounding all findings in participant data, with verbatim quotations provided throughout the Results to demonstrate the evidentiary basis for each theme and subtheme. Reflexivity processes documented throughout data collection and analysis provided transparency regarding the researchers’ role in interpretation.

### Ethical considerations

This study received ethical approval from the Standing Committee for Research Ethics at King Saud University (Reference: KSU-HE-25-1436), approved on 02 December 2025. The study was conducted in accordance with the principles of the Declaration of Helsinki. All participants provided written informed consent after receiving a bilingual (Arabic and English) information sheet explaining the study purpose, voluntary nature of participation, right to withdraw at any time without consequence, confidentiality protections, estimated interview duration (30–45 min), and data use restrictions. Confidentiality was protected through assignment of coded identifiers (P1–P17), secure storage of all data on password-protected institutional servers, and destruction of audio recordings after transcription verification. A pre-specified participant distress protocol was in place and followed during data collection. Should a participant display visible distress, the interviewer was to pause the recording, reconfirm consent, and provide Employee Assistance Programme (EAP) contact information. This protocol was invoked on one occasion, when a participant exhibited visible distress during discussion of a pediatric resuscitation event; the procedure was followed in full.

## Results

### Participant characteristics

The 17 participants demonstrated diverse demographic and professional profiles (Table 1). Ages ranged from 26 to 52 years (mean: 36.7; SD: 7.9). Total nursing experience ranged from 2 to 25 years (mean: 12.5; SD: 7.3), and ED-specific experience from 1 to 18 years (mean: 9.3; SD: 5.6). Nine participants held Bachelor of Science in Nursing (BSN) degrees and eight held diploma-level qualifications. Participants were distributed across two general hospitals (*n* = 10) and one specialized medical center (*n* = 7).

**Table 1:** Participant characteristics (*N* = 17)

ID	Age Group (years)	Total Nursing Experience (years)	ED Experience (years)	Highest Qualification	Facility Type
P1	30–39	10	7	BSN	General Hospital A
P2	20–30	4	2	Diploma	Specialized Center
P3	40–49	20	15	BSN	General Hospital A
P4	30–39	8	5	Diploma	General Hospital B
P5	51+	25	18	Diploma	Specialized Center
P6	20–30	2	1	BSN	Specialized Center
P7	30–39	15	10	BSN	General Hospital A
P8	30–39	6	4	Diploma	General Hospital B
P9	40–49	18	12	BSN	Specialized Center
P10	30–39	9	6	BSN	General Hospital A
P11	40–49	22	17	Diploma	General Hospital B
P12	20–30	5	3	BSN	Specialized Center
P13	30–39	13	9	BSN	General Hospital A
P14	30–39	11	8	Diploma	General Hospital B
P15	40–49	19	14	BSN	Specialized Center
P16	20–30	3	2	Diploma	Specialized Center
P17	40–49	23	16	Diploma	General Hospital B

BSN: Bachelor of Science in Nursing. Age categories are presented as ranges; actual ages ranged from 26 to 52 years. BSN *n* = 9 (53%); Diploma *n* = 8 (47%); General Hospital *n* = 10 (59%); Specialized Center *n* = 7 (41%). National and ethnic backgrounds were not collected; see Limitations

### Overview of themes

**Fig. 1:**
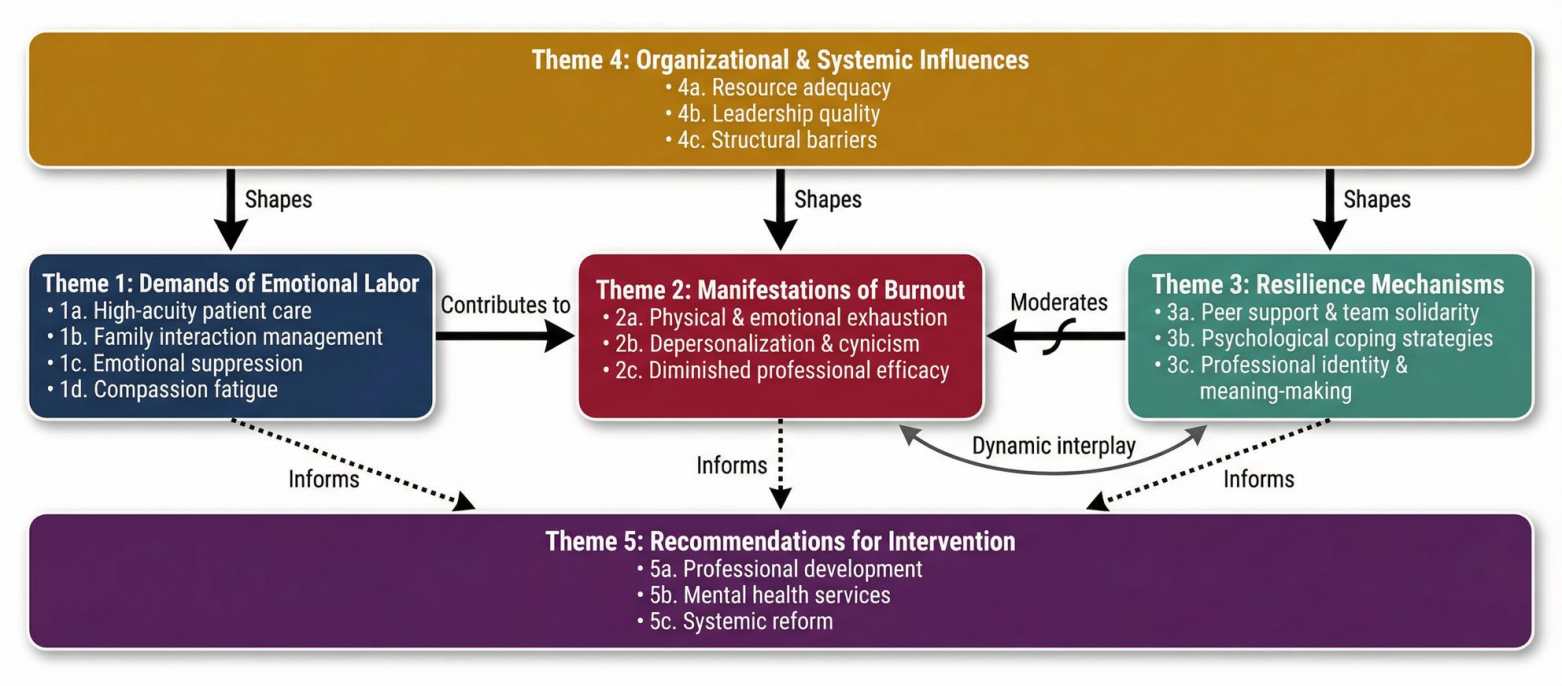
Thematic map illustrating relationships among five themes. Emotional labor demands (Theme 1) feed into burnout manifestations (Theme 2), which are moderated by resilience mechanisms (Theme 3). Organizational influences (Theme 4) shape all three processes. Recommendations (Theme 5) emerge from participants’ integrated experience across themes

Reflexive thematic analysis generated five themes and 16 subthemes (Table 2; Fig. 1). These themes are interconnected: emotional labor demands (Theme 1) contribute to burnout manifestations (Theme 2), which are modulated by resilience mechanisms (Theme 3), with organizational and systemic factors (Theme 4) influencing all three processes. Theme 5 represents participants’ own recommendations for intervention derived from their lived experience.

**Table 2:** Themes, subthemes, and descriptions

Theme	Subtheme	Description	Participants Endorsing
1. Demands of emotional labor	1a. High-acuity patient care	Psychological burden of rapid decision-making and sustained vigilance	17/17
	1b. Family interaction management	Managing anxious, distressed, or demanding family members	15/17
	1c. Emotional suppression	Conscious concealment of personal distress to maintain professional composure	16/17
	1d. Compassion fatigue	Cumulative emotional erosion from repeated trauma exposure	12/17
2. Manifestations of burnout	2a. Physical and emotional exhaustion	Persistent, restorative-resistant fatigue with somatic symptoms	15/17
	2b. Depersonalization and cynicism	Detached, objectifying attitudes toward patients	10/17
	2c. Diminished professional efficacy	Erosion of clinical confidence and self-doubt	11/17
3. Resilience mechanisms	3a. Peer support and team solidarity	Emotional sustenance, debriefing, and solidarity through collegial bonds	17/17
	3b. Psychological coping strategies	Mindfulness, physical activity, humor, compartmentalization, spiritual practice	16/17
	3c. Professional identity and meaning-making	Sustained purpose through sense of calling and patient care successes	14/17
4. Organizational influences	4a. Resource adequacy	Impact of staffing, equipment, and supply levels on stress and care quality	17/17
	4b. Leadership quality	Effect of managerial visibility, responsiveness, and advocacy	15/17
	4c. Structural barriers	Administrative burden, documentation overload, and inflexible policies	13/17
5. Recommendations	5a. Professional development	Structured education, mentorship, and career progression	14/17
	5b. Mental health services	Accessible, destigmatized counseling and debriefing	16/17
	5c. Systemic reform	Staffing optimization, workload reduction, and culture change	15/17

### Theme 1: Demands of emotional labor

All participants identified emotional labor as a central, pervasive dimension of ED practice. Four interconnected subthemes captured its manifestations.

#### Subtheme 1a: High-acuity patient care

Participants described the psychological weight of managing critically ill and injured patients under conditions of clinical uncertainty and acute time pressure. The sustained hypervigilance required depleted emotional resources alongside physical energy:

You’re managing a multi-trauma patient and making dozens of decisions in minutes. You have to be emotionally steady, but inside your mind is racing. It drains your emotions as much as your physical energy. (P3, 20 years’ experience)

This hypervigilance persisted beyond individual patient encounters. P9 (18 years’ experience) described difficulty disengaging from the alertness state: “By the end of the shift, even after the patient stabilises, your body doesn’t really relax. You carry it home.” Junior participants reported that uncertainty amplified the emotional burden: “When you’re not confident in your clinical decision, the emotional cost of every choice doubles” (P6, 2 years’ experience).

#### Subtheme 1b: Family interaction management

Managing family members’ distress was identified by 15 participants as a distinct and considerable source of emotional labor, frequently described as comparable in intensity to clinical care demands:

It’s often harder to manage the family’s panic than the patient’s condition. You have to be this calm anchor, absorbing their stress while doing your job. (P7, 15 years’ experience)

Several participants connected this demand to cultural expectations. Within Saudi collectivist culture, family involvement in healthcare is both expected and valued, which amplifies the emotional labor of the nurse–family interface: “When family members are frightened, they sometimes direct anger at us. We have to remain professional, as that emotional regulation is exhausting” (P5, 25 years’ experience). P14 (11 years’ experience) noted: “In our culture, families want to be involved in everything. You’re not just caring for one patient; you’re managing an entire family’s emotions.”

#### Subtheme 1c: Emotional suppression

Sixteen participants described the habitual concealment of personal distress as a learned professional skill that, paradoxically, consumed substantial psychological resources:

Inside you’re breaking after a pediatric code, but outside you’re steady. You can’t let them see you crumble, because it’s part of the uniform. (P11, 22 years’ experience)

This surface acting, characterized by maintaining an outward display inconsistent with inner emotional states, was recognized by participants as progressively more effortful over time. P10 (9 years’ experience) reflected: “Early on, you think suppressing emotions is professional strength. Over time, you realize it’s costing you.” 

#### Subtheme 1d: Compassion fatigue

Several participants, predominantly those with longer tenure, described gradual erosion of empathic capacity through cumulative trauma exposure:

Sometimes I feel I’ve seen so much suffering that I’m becoming numb. Early in my career, every loss affected me deeply. Now I can discuss a patient’s death factually and realize I’m not feeling the sadness I used to. (P14, 11 years’ experience)

Notably, this emotional numbing was experienced with discomfort rather than acceptance, suggesting self-awareness of compassion fatigue as a concerning occupational consequence rather than a desired professional adaptation. P3 (20 years’ experience) described this tension: “I notice it happening and it worries me. It’s not who I want to be as a nurse.”

### Theme 2: Manifestations of burnout

Burnout was described not as a discrete event but as a progressive process that emerged when emotional demands chronically outpaced available personal and organizational resources. Most participants described at least one burnout dimension; several described manifestations across all three subthemes.

#### Subtheme 2a: Physical and emotional exhaustion

Participants described fatigue extending beyond expected work tiredness into persistent, restorative-resistant depletion: “I wake up tired. It’s a bone-deep, ongoing exhaustion that sleep doesn’t fix” (P9, 18 years’ experience). The cumulative nature was emphasized, as noted by P4: “It’s not one difficult shift; it’s accumulation. Your body never fully recovers before the next one begins” (P4, 8 years’ experience). Several participants connected exhaustion to physical manifestations including chronic headaches, insomnia, and gastrointestinal symptoms.

#### Subtheme 2b: Depersonalization and cynicism

Several participants acknowledged developing objectifying or negative attitudes toward patients that conflicted with their professional values:

I find myself seeing patients as ‘another admission’ instead of people. It’s a defence mechanism, but it feels awful to lose that connection. (P13, 13 years’ experience)

This self-awareness, characterized by the recognition of cynicism as ego-dystonic and incongruent with one’s professional identity, was a notable and recurring feature, suggesting that depersonalization was experienced as a troubling defensive adaptation rather than a stable attitudinal shift. P8 (6 years’ experience) described: “I catch myself being dismissive and it shocks me. That’s not who I am.”

#### Subtheme 2c: Diminished professional efficacy

Several participants, particularly those earlier in their ED careers, described erosion of clinical confidence: “I second-guess every decision. You feel like you’re just not good enough anymore” (P6, 2 years’ experience). Importantly, this dimension was closely linked to organizational resource constraints: several participants attributed their self-doubt partly to systemic conditions that prevented them from delivering care consistent with their professional standards. P15 (19 years’ experience) articulated this connection: “It’s not that I’ve lost my skills. It’s that the system doesn’t let me use them properly.”

### Theme 3: Resilience mechanisms

Despite the substantial demands described above, all participants employed diverse adaptive strategies that sustained their professional engagement and psychological functioning. Resilience manifested not as an absence of distress but as adaptive functioning despite distress.

#### Subtheme 3a: Peer support and team solidarity

Collegial relationships were universally identified as the single most potent resilience factor. Peer support operated simultaneously at emotional (validation, shared grief), practical (workload distribution), and identity-affirming (shared professional understanding) levels:

My team is my lifeline. We debrief after a bad code, just talking it through. Knowing they truly get it makes the unbearable moments bearable. (P3, 20 years’ experience)

Team cohesion was described as multiplicative in its protective effects: “When we feel cohesive, even intense shifts feel manageable. When team dynamics fracture, everything becomes harder” (P14, 11 years’ experience). The centrality of this mechanism aligns with the collectivist cultural orientation of Saudi society, where group solidarity and relational bonds carry particular cultural weight. P17 (23 years’ experience) reflected: “In our culture, we lean on each other. That’s how we survive.”

#### Subtheme 3b: Psychological coping strategies

Participants employed diverse, individually adapted approaches including mindfulness (“even five minutes of focused breathing between patients helps me reset,” P12, 5 years’ experience), physical activity (“running after a difficult shift helps me physically process the stress,” P15, 19 years’ experience), humor (“dark humor within the team, which outsiders might not understand, but it’s our way of processing impossible situations,” P7, 15 years’ experience), compartmentalization (“I’ve learned to separate work from home. When I leave the department, I consciously leave it behind,” P10, 9 years’ experience), and spiritual practice. Several participants specifically referenced prayer and religious faith as coping resources: “Prayer gives me perspective. It reminds me that suffering has meaning in God’s plan, and that helps me find peace after difficult cases” (P9, 18 years’ experience). The prominence of spiritual coping reflects the centrality of Islamic faith in Saudi daily life and its documented role in psychological adaptation.

#### Subtheme 3c: Professional identity and meaning-making

Most participants described sustained sense of professional purpose as a powerful resource that buffered against burnout progression:

Remembering why I became a nurse helps. The small wins, such as patients who get better, families who feel heard, are what sustains me. (P11, 22 years’ experience)

Senior participants described meaning-making as wisdom accumulated through career experience: “You learn what you can control and what you cannot. You find satisfaction in small victories” (P5, 25 years’ experience).

### Theme 4: Organizational and systemic influences

Organizational factors were identified by all participants as substantially shaping both burnout trajectories and resilience capacity, and participants consistently located these factors as more influential than individual coping in determining occupational wellbeing.

#### Subtheme 4a: Resource adequacy

Inadequate staffing, equipment, and supplies were universally cited as stressors that compounded emotional labor demands and generated moral distress:

It’s impossible to give good care when you’re short-staffed and hunting for a working IV pump. It creates immense frustration, you know what quality care looks like, but the system prevents you from providing it. (P8, 6 years’ experience)

P15 (19 years’ experience) framed resource inadequacy as a systemic rather than individual issue: “Burnout isn’t about emotional weakness. It’s about being inadequately resourced to meet impossible demands.” P3 (20 years’ experience) articulated the moral distress this generated: “You’re making compromises about care quality constantly. That’s morally injurious.”

#### Subtheme 4b: Leadership quality

The quality of nurse–manager relationships was identified by most participants as pivotal to their occupational experience. Supportive leaders who demonstrated visibility, responsiveness, and advocacy created conditions for resilience: “A manager who truly listens and advocates for our needs to administration makes a tremendous difference” (P9, 18 years’ experience). Conversely, distant or unresponsive leadership compounded isolation and demoralization: “When concerns aren’t addressed and there’s no acknowledgement of what we carry, you feel invisible” (P4, 8 years’ experience). P14 (11 years’ experience) described the protective mechanism: “A leader who rounds in the ED, who asks how we’re really doing, that creates a culture of psychological safety.” 

#### Subtheme 4c: Structural barriers

Several participants identified administrative burden, excessive documentation requirements, and inflexible organizational policies as diverting time from patient care and contributing to frustration: “We spend enormous time charting that doesn’t directly benefit patients. I become a computer operator rather than a nurse” (P7, 15 years’ experience). P12 (5 years’ experience) described policies misaligned with clinical reality: “Rigid policies don’t account for clinical complexity. You’re trying to make individualized decisions, but compliance requirements constrain clinical judgment.”

### Theme 5: Recommendation for intervention

Participants articulated specific, experience-grounded recommendations organized around three domains.

#### Subtheme 5a: Professional developments

Most participants advocated for protected time for continuing education, structured mentorship programs pairing junior nurses with experienced colleagues, and expanded career progression pathways including clinical specialist, educator, and research roles. P6 (2 years’ experience) described: “More structured mentorship and gradual advancement into high-acuity care would build confidence and reduce that sense of being thrown into the deep end.” P17 (23 years’ experience) emphasized that career progression sustains long-term engagement: “Pathways beyond bedside nursing, education, leadership, help you stay in the profession as physical demands accumulate.”

#### Subtheme 5b: Mental health services

Most participants emphasized the need for accessible, confidential, destigmatized counseling staffed by professionals familiar with ED culture and demands: “There should be dedicated, confidential counseling on-site, not requiring us to seek it out, but proactively reaching out after critical incidents” (P9, 18 years’ experience). Normalization was emphasized: “Seeking support shouldn’t carry stigma. Regular wellbeing check-ins should be part of the culture, not an exception” (P14, 11 years’ experience).

#### Subtheme 5c: Systemic reform

Most participants called for enforceable safe staffing ratios, reduction of non-essential documentation, and organizational culture transformation prioritizing staff wellbeing: “We need real changes, better staffing ratios and a culture that values us as people, not just as workers filling shifts” (P5, 25 years’ experience). P7 (15 years’ experience) advocated for frontline input: “Workflows should be designed with input from the people who actually work in the ED. Policies made by people disconnected from emergency practice don’t serve us.”

## Discussion

This study provides a contextually grounded qualitative account of how ED nurses in Saudi Arabia experience the interplay between emotional labor, burnout, and resilience. Four key findings warrant discussion within the broader theoretical and empirical literature.

### Burnout and resilience as concurrent processes

Perhaps the most significant finding is that burnout and resilience were not experienced as mutually exclusive states occupying opposite poles of a single continuum. Rather, participants described the concurrent presence of burnout manifestations, including exhaustion, cynicism, diminished efficacy, alongside active resilience mechanisms including peer support, meaning-making, and coping strategy deployment. Participants who described significant exhaustion simultaneously maintained professional meaning; those experiencing cynicism retained awareness that their attitudes were incongruent with their professional values and actively sought to counteract them.

This co-occurrence challenges traditional conceptualizations that position burnout and resilience as inversely related constructs [4, 6] and aligns with emergent theoretical models recognizing their coexistence as distinct, co-occurring processes [29, 30]. Through the lens of COR theory [18], this finding is mechanistically coherent: COR theory’s domain-specific resource architecture explains: burnout arises from net resource loss in emotionally depleting domains (affective energy, physical reserves), while resilience is maintained through resource conservation or gain in parallel but non-identical domains (collegial bonds, professional identity, spiritual capital). A nurse can simultaneously experience emotional exhaustion, a domain-specific deficit, and robust peer solidarity, a domain-specific surplus; the two processes co-occur across separate resource pools rather than cancelling each other. The clinical and policy implication is consequential: the presence of resilience indicators in a workforce should not be interpreted as evidence that burnout is absent, and interventions must address both processes concurrently rather than assuming that strengthening resilience will automatically prevent burnout.

### Emotional labor as under-recognized occupational work

Participants described emotional labor as pervasive and progressively depleting, consistent with Hochschild’s [3] foundational theory and with nursing-specific evidence [4, 31]. Emotional suppression, the conscious concealment of personal distress to maintain professional composure, was identified as particularly costly to psychological resources, functioning as a form of surface acting that accumulated emotional debt over time. This aligns with meta-analytic evidence linking surface acting to burnout across occupational contexts [32] and extends it by demonstrating how specific cultural demands amplify these costs within the Saudi context.

Notably, the cultural expectation of family involvement in healthcare created a distinct layer of emotional labor not typically foregrounded in Western studies. Participants described managing entire family systems’ emotions alongside clinical care, a dual emotional burden reflecting Saudi collectivist values wherein the family unit, rather than the individual patient alone, constitutes the focus of care [20, 21]. This culturally specific dimension of emotional labor warrants recognition in organizational policy and training programs designed for Middle Eastern healthcare contexts.

A further notable finding was that participants experienced their developing compassion fatigue and emotional numbing with explicit discomfort and self-awareness. This suggests that emotional labor awareness, the metacognitive recognition of one’s emotional work as legitimate occupational labor rather than a natural extension of caring, may itself function as a protective factor, enabling targeted coping rather than passive depletion. This interpretation aligns with emerging evidence suggesting that emotional intelligence and emotional labor awareness moderate the relationship between emotional demands and burnout outcomes [33].

### The primacy of organizational context

While individual coping strategies were diverse and clearly valued, participants consistently and emphatically located the primary determinants of burnout and resilience at the organizational rather than the individual level. Resource inadequacy, leadership quality, and structural barriers were described as more influential than personal coping capacity in determining whether nurses experienced sustained wellbeing or progressive burnout. This finding directly challenges intervention approaches that focus predominantly on building individual resilience without addressing the organizational conditions generating occupational demands.

From a COR perspective [18], organizational factors constitute the resource ecology within which individual resource management occurs. When the organizational environment is chronically depleting, through inadequate staffing, absent leadership support, excessive administrative burden, and inflexible policies, individual coping strategies become insufficient to prevent net resource loss, regardless of their sophistication. This interpretation aligns with critical scholarship arguing that individually-focused resilience programs risk shifting responsibility for adaptation onto workers rather than addressing the systems that generate unsustainable demands [34, 35].

The practical implication is that organizational-level interventions, including staffing optimization, leadership development emphasizing emotional intelligence and staff advocacy, administrative burden reduction, and systemic culture change, should be positioned as foundational, with individual support strategies as complementary rather than primary. P15’s statement that “burnout isn’t about emotional weakness; it’s about being inadequately resourced to meet impossible demands” encapsulates this organizational accountability frame.

### Cultural specificity and intervention design

Findings revealed culturally specific mechanisms that constitute a distinct analytical contribution of this study, not merely contextual background. Three mechanisms differentiate this Saudi sample from predominantly Western populations. The primacy of peer support as a resilience mechanism aligns with collectivist cultural values that prioritize group solidarity and relational interdependence over individual self-reliance [21]. The specific emotional labor generated by culturally expected family involvement in healthcare represents a demand dimension inadequately captured by emotional labor instruments developed in Western contexts [3]. The prominence of Islamic spiritual practice, including, prayer, faith-based meaning-making, and trust in divine purpose, as a coping resource reflects the centrality of religion in Saudi daily life and its documented role in psychological adaptation among Arab populations [36].

These findings carry important implications for intervention design. Programs developed in Western, secular, individualist healthcare contexts cannot be uncritically imported into Saudi settings. Effective interventions must be culturally congruent, incorporating family management skills training, leveraging the protective effects of team collectivism rather than emphasizing individual self-care alone, and acknowledging spiritual coping as a legitimate and valued dimension of resilience. Future intervention research should employ participatory design processes that include Saudi ED nurses as co-designers rather than solely as recipients of externally developed programs.

### Implications for practice and policy

Findings support a multi-level intervention framework:

At the *individual* level, evidence-based stress management training incorporating emotional labor awareness, mindfulness, coping flexibility, and the validation of spiritual coping strategies should be offered through accessible, protected-time programs.

At the *interpersonal* level, formalized peer support programs and structured post-incident debriefing protocols should be established, leveraging the potent protective effects of team cohesion documented across all participants.

At the *organizational* level, interventions should target safe staffing ratios based on patient acuity, leadership development programs emphasizing emotional intelligence and visible staff advocacy, reduction of non-essential administrative documentation, establishment of accessible and destigmatized on-site mental health services, and systematic organizational culture change embedding staff wellbeing as a strategic priority.

At the *policy* level, occupational health standards specific to ED nursing should be developed and integrated into Saudi healthcare accreditation frameworks, with mandated organizational reporting on nurse wellbeing indicators alongside traditional patient outcome metrics.

### Strengths and limitations

This study possesses several methodological strengths. The multi-site design encompassing three facilities with different organizational profiles enhanced participant diversity and transferability of findings. Multiple trustworthiness strategies, including member checking with 35% of participants, peer debriefing across a five-member research team with complementary expertise, a comprehensive audit trail, and systematic reflexive journaling, provided robust mechanisms for ensuring credibility, dependability, and confirmability. The explicit constructivist epistemological positioning, dual theoretical framework (COR theory, emotional labor theory), and transparent analytical process (reflexive thematic analysis with documented code development) demonstrate methodological coherence. The study addresses a significant gap in the qualitative literature on ED nurse wellbeing in Middle Eastern contexts, where quantitative prevalence studies have predominated.

Several limitations warrant acknowledgment. The sample of 17 nurses from three urban facilities in Riyadh, while appropriate for qualitative inquiry and consistent with information power principles [29], limits transferability to rural Saudi settings or healthcare systems with substantially different organizational structures. Although 23 nurses expressed interest and 17 participated, the two who declined cited scheduling constraints; it remains possible that nurses experiencing the most severe burnout were underrepresented due to energy or time limitations that precluded participation. Despite reflexive strategies, the insider positionality of all team members, who all hold nursing qualifications, may have influenced interpretation toward professional norms. The cross-sectional interview design captured retrospective accounts at a single time point, precluding examination of how burnout–resilience dynamics evolve longitudinally. Participant national and ethnic backgrounds were not collected; this omission may limit the extent to which culture-specific interpretations can be attributed solely to the Saudi healthcare context, and readers should exercise caution in generalising findings. No systematic thematic differences were observed between participants interviewed in Arabic and those interviewed in English; however, the unequal distribution across language groups (12 Arabic, 5 English) may have introduced unmeasured variation. The predominantly Arabic-language interviews required translation for reporting, introducing potential for nuance loss despite the verification procedures employed.

## Conclusions

This qualitative study demonstrates that ED nurses in Saudi Arabia navigate a complex, concurrent interplay between emotional labor demands, burnout manifestations, and resilience mechanisms. Burnout and resilience coexist as dynamic processes rather than opposing endpoints, and organizational context, particularly resource adequacy, leadership quality, and access to mental health support, substantially determines whether individual adaptive capacities translate into sustained occupational wellbeing. The culturally specific dimensions of emotional labor, peer support, and spiritual coping identified in this study underscore the importance of context-sensitive intervention design.

These findings reinforce an organizational accountability imperative: the burden of adaptation must not fall solely on individual nurses. Healthcare organizations bear ethical and operational responsibility for creating conditions that enable sustainable emergency nursing practice, with staffing adequacy, leadership responsiveness, and destigmatized access to mental health support as foundational systemic requirements. Future longitudinal research should examine how burnout–resilience dynamics evolve across career trajectories and evaluate the effectiveness of culturally congruent, multi-level organizational interventions within the Saudi Arabian and broader Middle Eastern healthcare context.

## Data Availability

The qualitative datasets generated during the current study are not publicly available due to the nature of the informed consent provided by participants and the potential for identification through detailed narrative data. Anonymized excerpts may be available from the corresponding author upon reasonable request, subject to ethical review.

## Abbreviations

BSN: Bachelor of Science in Nursing
COR: Conservation of Resources
COREQ: Consolidated Criteria for Reporting Qualitative Research
ED: Emergency Department
SD: Standard Deviation
